# Serum Biochemical Parameters, Rumen Fermentation, and Rumen Bacterial Communities Are Partly Driven by the Breed and Sex of Cattle When Fed High-Grain Diet

**DOI:** 10.3390/microorganisms10020323

**Published:** 2022-01-30

**Authors:** Xinjun Qiu, Xiaoli Qin, Liming Chen, Zhiming Chen, Rikang Hao, Siyu Zhang, Shunran Yang, Lina Wang, Yafang Cui, Yingqi Li, Yiheng Ma, Binghai Cao, Huawei Su

**Affiliations:** 1 State Key Laboratory of Animal Nutrition, College of Animal Science and Technology, China Agricultural University, Beijing 100000, China; qiuxinjun@cau.edu.cn (X.Q.); qinxiaolicau@outlook.com (X.Q.); chenzhim@cau.edu.cn (Z.C.); haork@cau.edu.cn (R.H.); s20213040648@cau.edu.cn (S.Z.); sy20213040761@cau.edu.cn (S.Y.); linawang@cau.edu.cn (L.W.); s20203040609@cau.edu.cn (Y.C.); liyingqi1230@163.com (Y.L.); mayihengabc@cau.edu.cn (Y.M.); 2 Hubei Fulljoywo Agricultural Development Company Limited, Yichang 443000, China; clm191213@163.com

**Keywords:** breed, fattening performance, rumen bacteria, rumen fermentation, serum parameters, sex

## Abstract

Hybridization in bovines is practiced with the main aim of improving production performance, which may imply the microbial variations in the rumen from the parental breed cross to their progeny. Besides, the interactions of offspring breed with sex in terms of rumen bacteria are not clear. This study aims to evaluate the variations in rumen bacterial communities in different breeds and sexes, and the correlations among fattening performance, serum biochemical parameters, and rumen fermentation. Forty-two 19.2 ± 0.67-month-old beef cattle (390 ± 95 kg of initial body weight) comprising two genetic lines (Yiling and Angus × Yiling) and two sexes (heifers and steers) were raised under the same high-grain diet for 120 d. On the last two days, blood samples were collected from each animal via the jugular vein before morning feeding for analyzing serum biochemical parameters; rumen fluid samples were obtained via esophageal intubation 2 h after morning feeding for analyzing rumen fermentation parameters and bacterial communities. The results show that both breed and sex had a certain impact on fattening performance, serum biochemical parameters, and rumen fermentation. No differences in the diversity and structure of rumen bacterial communities were observed. Significant interactions (*p* < 0.05) of breed and sex were observed for *Succinivibrionaceae UCG-002* and *Prevotellaceae UCG-001*. The relative abundances of the *Rikenellaceae RC9 gut group*, *Prevotellaceae UCG-003*, and *Succinivibrio* were different (*p* < 0.05) between breeds. Heifers had a higher (*p* = 0.008) relative abundance of the *Rikenellaceae RC9 gut group* than steers. Correlation analysis showed a significant relationship (*p* < 0.05) of rumen bacteria with serum biochemical parameters, rumen pH, and rumen fermentation patterns. Additionally, only two genera, *Prevotellaceae UCG-003* and *Prevotellaceae UCG-001*, had positive correlations with feed efficiency. In conclusion, serum biochemical parameters, rumen fermentation, and rumen bacterial communities are partly driven by the breed and sex of cattle fed a high-grain diet.

## 1. Introduction

Fifty-five indigenous bovine breeds with nearly 30 million animals have been observed in China [1]. In general, they are characterized by a small size, slow growth, inferior dressing percentage, etc. These characteristics have hindered the current beef market. The Yiling (YL) breed is typically raised in the Yichang district, Hubei province. It was formerly selected as a draught animal, but now this breed and its hybrids are bred only for beef production, especially for high marbling beef. A previous study [2] evaluated its genetic background. Furthermore, the finishing performance of YL cull cows was evaluated in our previous feeding experiment [3]. As expected, it presented an inferior performance in daily gain and feed efficiency. In order to protect and utilize this genetic resource, YL cattle and its hybrids need to be further evaluated to provide a scientific basis for its breeding and industrialization.

Ruminants have evolved a complex and diverse symbiotic microbiota consisting of bacteria, archaea, protozoa, fungi, and viruses in their rumen [4]. In particular, rumen bacteria are the most abundant microbiota in terms of diversity and account for the vast majority of the microbiome. Additionally, they have been the focus of most quantitative studies on rumen microbial composition. These microbes of ruminants can typically degrade plant fibers and utilize non-protein nitrogen to produce volatile fatty acids (VFAs) and microbial proteins, further meeting the host’s nutrient requirements for maintenance and production. Our recent efforts indicated that the differences in rumen bacterial communities were particularly associated with diet, including forage inclusion [5,6], energy levels [7], protein levels [8], and even nutrient density [9]. These studies also confirmed the correlations of rumen bacterial abundances with the host’s phenotypic characteristics, such as nutrient intake [6,9], rumen fermentation products [6,7,9], nutrient apparent digestibility [6,9], blood metabolites [6,9], and meat fatty acids [7]. Rumen microbiota contributes to the host’s nutrient availability and subsequently exerts a potential impact on production performance. In this sense, it is effective to establish the interactions among diet, rumen microbiota, and phenotypic characteristics in ruminants. Recent genome-wide association studies revealed that the composition of rumen microbiota can be affected by host additive genetics or genotypes at multiple taxonomic levels [10,11,12]. Furthermore, heritable rumen microbial features are associated with rumen metabolites [10,12,13], feed efficiency [10], and milk quality [12,13]. These observations more strongly confirm another notion of a triangular relationship among the host genetics, rumen microbiota, and phenotypic characteristics.

Several studies have explored the rumen microbial differences driven by different cross combinations. For instance, Li et al. [14] reported that the microbiota and metabolites in the rumen were largely affected by different hybrid crosses between sika deer and elk; Bainbridge et al. [15] reported that rumen bacterial communities were less affected by dairy breeds (Holstein, Jersey, and Holstein × Jersey) when compared with lactation days; Hernandez-Sanabria et al. [16] and Roehe et al. [17] found a significant effect of sired beef breed on rumen bacterial and archaeal communities. These results imply the potential differences in rumen bacterial communities between purebred and crossbred breeds. However, the interaction with sex was not explored in these studies.

Here, we compared the differences in rumen bacterial communities driven by the breed (YL vs. Angus × YL (AY)) and sex (steers vs. heifers) of cattle fed the same high-grain diet. We also analyzed the correlations of rumen bacterial communities with fattening performance, serum biochemical parameters, and rumen fermentation. We hypothesized that breed may impact the fattening performance and bacterial communities of cattle regardless of sex. It should be noted that this breed factor can also be further defined as a different sired breed (YL vs. Angus) in the current study. Thus, the improvement in fattening performance and even the differences in the rumen bacterial communities of crossing progenies mainly derive from the transmission of superior traits from sires [16]. In this sense, the particular rumen bacterial communities of crossing progenies could be used as a reference for improving the productivity of Chinese indigenous cattle.

## 2. Materials and Methods

### 2.1. Ethics Statement

Animal studies were performed in accordance with institutional guidelines and the approval of the Animal Care and Use Committee of China Agricultural University (Permit No. AW09059102-3, 6 September 2017).

### 2.2. Animals, Management, and Sampling

Before the trial, all cattle were weaned at 4 months old and castrated at 5 months old and subsequently received the same diet and management. Forty-two cattle aged 19.2 ± 0.67 months were selected and fed the same total mixed ration (TMR) for the 120 d fattening trial. These cattle comprised two genetic lines and two sexes: YL heifers (*n* = 10) and steers (*n* = 10); Angus × YL (AY, sire × dam) heifers (*n* = 9), and steers (*n* = 13). All animals were reared in separate pens with ad libitum access to TMR based on 5 to 10% orts. Experimental TMR (11.4 MJ/kg metabolic energy, 12.0% crude protein) was formed by 20.0% corn silage, 6.63% rice straw, and 73.3% concentrate composed of corn grain, wheat bran, rapeseed cake, soybean meal, limestone, premix, NaHCO_3_, and NaCl. The feeding frequency of TMR was twice a day at 08:00 h and 16:00 h.

Feed provided and residue were recorded daily to calculate the average dry matter intake (DMI) during the whole fattening period. Body weight (BW) was recorded before morning feeding for 3 consecutive days. Average daily gain (ADG) was calculated based on the difference between initial body weight and final body weight. The last 7 days of the fattening period were the sampling phase. Blood samples were collected from each animal via the jugular vein before morning feeding and then centrifuged at 3500× *g* for 10 min to obtain serum and subsequently stored at −80 °C until serum biochemical parameters analysis. A total of 41 rumen fluid samples were collected via esophageal intubation 2 h after morning feeding (discarded one sample polluted by saliva). The first 200 mL of rumen fluid samples was discarded to minimize contamination from the saliva. The pH value of rumen fluid samples was measured immediately. Then, rumen fluid samples were filtered using four layers of sterile cheesecloth. Two aliquots were stored at −80 °C for VFA and ammonia-N analysis, respectively. Another two aliquots were stored at −80 °C for 16S rRNA pyrosequencing.

### 2.3. Chemical Analysis

Glucose (GLU), triglyceride (TG), cholesterol (CHO), non-esterified fatty acid (NEFA), beta-hydroxybutyrate (BHB), high-density lipoprotein cholesterol (HDL-C), low-density lipoprotein cholesterol (LDL-C), creatinine (CREA), urea (UREA), aspartate aminotransferase (AST), alanine aminotransferase (ALT), alkaline phosphatase (ALP), total protein (TP), and albumin (ALB) concentrations were determined using commercial test kits (Beijing Jiuqiang Bio-Technique Co. Ltd., Beijing, China) with an automated biochemistry analyzer (Hitachi 7020; Hitachi Ltd., Tokyo, Japan). The ammonia-N concentration of rumen liquid was measured with a spectrophotometer (UV-1700, Shimadzu Corporation, Kyoto, Japan) according to the method described by Weartherburn [18]. The VFAs of rumen liquid were quantified using high-performance gas chromatography (HPGC; GC-2014; Shimadzu Corporation) equipped with a hydrogen flame detector and a capillary column (Agilent Technologies, Inc., Wilmington, DE, USA; 30 m long, 0.32 mm diameter, 0.50 µm film).

### 2.4. DNA Extraction, PCR Amplification, and MiSeq Sequencing

The DNA extraction, PCR amplification, and MiSeq sequencing of 41 rumen liquid samples were outsourced to Allwegene Company (Beijing, China). The DNA was extracted from rumen fluid samples using the bacterial DNA Kit (Omega Bio-Tek Inc., Norcross, GA, USA). The DNA concentration and purity were preliminarily evaluated by using the Shimadzu spectrophotometer. The hypervariable V3-V4 region of bacterial 16S rRNA genes was amplified from extracted DNA using the barcoded primers 338F (5′-ACTCCTACGGGAGGCAGCAG-3′) and 806R (5′-GGACTACHVGGGTWTCTAAT-3′). All PCR reactions consisted of 30 ng of extracted DNA sample, 2 μL of forward primer and reverse primer (10.0 μmol/L), 4 μL of DNA template (2.5 μmol/L), 36.7 μL of RNase-free ddH_2_O, 5 μL of 10 × Pyrobest Buffer, and 0.3 μL of Pyrobest DNA Polymerase (2.5 U/μL, TaKaRa Code: DR005A). The thermal cycling procedures were as follows: 5 min of denaturation at 95 °C, followed by 25 cycles of 30 s for denaturation at 95 °C, 30 s for annealing at 56 °C, and 40 s for elongation at 72 °C, along with a final extension at 72 °C for 10 min. The amplified PCR products were analyzed by 1% agarose gel electrophoresis and purified using Agencourt AMPure XP kit (Becker Coulter, Inc., Brea, CA, USA). Purified amplicons were pooled in equimolar and paired-end sequenced on an Illumina MiSeq platform (Illumina, Inc., San Diego, CA, USA).

### 2.5. Bioinformatic Analysis

The raw data in FASTQ files were processed and quality-filtered using Trimmomatic (v0.36) and PEAR (v0.9.6). Specifically, reads were removed if they were shorter than 260 bp, had a quality score below 20, or had ambiguous bases. Paired-end reads were assembled using FLASH (v1.20) and PEAR with the following assembly parameters: 10 bp of minimal overlapping and 10% of maximum mismatch rate. USEARCH (v2.7.1) was used to remove chimeric sequences based on the UCHIME algorithm. USEARCH then clustered the sequences into individual OTUs at 97% identity. The representative sequences for each OTU were picked using Quantitative Insights Into Microbial Ecology (QIIME v1.8), which were assigned from the SILVA database. Rarefaction curves were analyzed using MOTHUR. Alpha diversity was determined using Shannon and Chao1 indices and calculated using procedures within QIIME. Principal coordinate analysis (PCoA) and permutational multivariate analysis of variance (PERMANOVA) were performed based on Bray-Curtis dissimilarity matrix using vegan package (v2.5-7) of R (v4.1.2). Correlation analysis and hierarchical clustering were performed using Spearman’s rank correlation and complete linkage method, respectively. The results of PCoA and correlation analysis were visualized using ggplot2 (v3.3.5) and pheatmap package (v1.0.12), respectively. The *p* values from correlation analysis and PERMANOVA lower than 0.05 were considered significant, while *p* values less than 0.05 and absolute value of correlation coefficients more than 0.3 were regarded as significant correlations.

### 2.6. Statistics Analysis

Statistical analyses were conducted using the software SAS (SAS Inst. Inc., Cary, NC, USA). Data were analyzed using a general linear model in a factorial 2 × 2 arrangement with four groups:

Y_ij_ = μ + B_i_ + G_j_ + (BG)_ij_ + e_ij_,

where Y_ij_ is the dependent variable, μ is the overall mean, B_i_ is the effect of breed, G_j_ is the effect of sex, (BG)_ij_ is the interaction of breed and sex, and e_ij_ is the residual error. The least squares means were compared using LSD post hoc test when the interaction was significant. Statistical differences were declared significant at *p* ≤ 0.05.

## 3. Results

### 3.1. Fattening Performance

The fattening performance for all cattle across the entire fattening trial is shown in Table 1. The initial BW of AY crossbred cattle was higher (*p* < 0.001) than that of YL purebred cattle. Heifers had lower (*p* < 0.001) initial BW compared to steers, regardless of the breed. The interactions of breed and sex were observed (*p* < 0.05) for final BW and ADG. Multiple comparisons revealed that YL heifers had lower (*p* = 0.003) final weight than YL steers, whereas AY heifers had higher (*p* < 0.001) ADG than AY steers. Besides, AY cattle had higher DMI than YL cattle. However, the feed conversion rate was unaffected by breed and sex.

### 3.2. Serum Biochemical Parameters

The results show that the interactions of breed and sex were statistically insignificant (*p* > 0.05) for all serum biochemical parameters (Table 2). Considering the main effect, AY cattle had higher (*p* = 0.025) AST levels and lower (*p* < 0.001) ALP levels compared to YL cattle. In addition, the GLU, BHB, and ALP levels of steers were greater (*p* < 0.05) than those of heifers, regardless of breed.

### 3.3. Rumen Fermentation

The interaction of breed and sex was significant (*p* = 0.012) for ammonia-N concentration in the rumen (Table 3). The rumen pH value-to-acetate propionate ratio was significantly lower (*p* < 0.05) in AY cattle than in YL cattle, regardless of sex. In addition, steers had higher (*p* < 0.05) isobutyrate and valerate concentrations than heifers, regardless of breed.

### 3.4. Bacterial Diversity and Composition

A total of 5,092,309 valid sequences, with an average of 124,203 ± 11,932 sequences per sample, were retained after quality control and chimaera removal. Furthermore, 99.96% of sequences were distributed between the lengths of 400 bp and 440 bp. A total of 4981 OTUs (1783 ± 42.0 per sample) were observed based on 97% sequence similarity. The percentage of Good’s coverage was determined with a mean value of 98.3% across all samples, indicating sufficient sequence coverage for all samples. The Chao1, Shannon, Simpson, and phylogenetic diversity (PD) of whole-tree indexes were measured to compare the alpha bacterial diversity within the four groups (Table 4), and all indexes were unaffected by both breed and sex.

**Figure 1:**
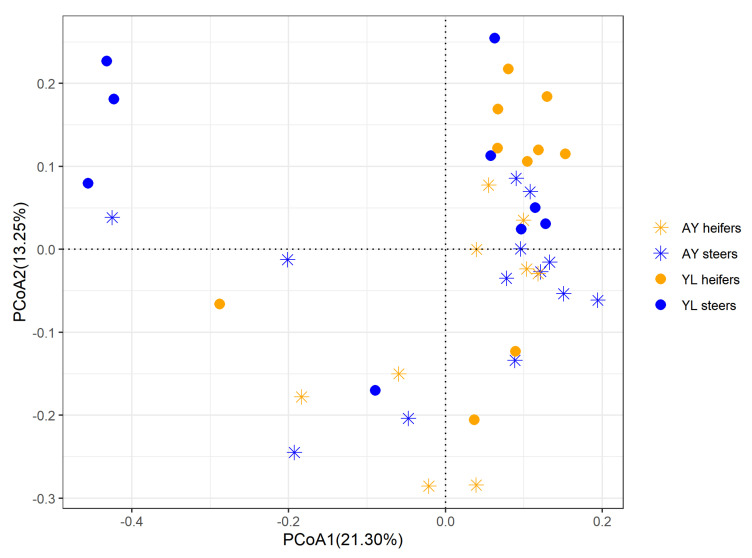
Principal coordinates analysis (PCoA) plot based on Bray-Curtis dissimilarity matrix to compare the bacterial structure among Angus × Yiling (AY) heifers, AY steers, Yiling (YL) heifers, and YL steers.

The PCoA analysis was performed to evaluate the beta diversity of the bacterial communities based on the Bray-Curtis distance matrix (Figure 1). The clustering of bacteria from the four groups overlapped, and hence no clear distinction was noticed from the PCoA analysis. Furthermore, PERMANOVA also showed that the effects of breed (R^2^ = 0.652, *p* = 0.920) and sex (R^2^ = 0.742, *p* = 0.179) and their interaction (R^2^ = 0.664, *p* = 0.188) on the structure of the bacterial communities was not significant.

At the phylum level, a total of 10 bacteria phyla with an average relative abundance ≥ 0.1% were detected (Table 5). *Bacteroidetes* (56.0–64.6%) followed by *Firmicutes* (29.3–38.6%) were the largest bacterial phyla in four groups, together representing 92.8% of all bacteria, and these two phyla did not differ (*p* > 0.05) between all groups. No significant interactions (*p* > 0.05) of breed and sex were observed for any phyla except for *Proteobacteria* (*p* = 0.033), which was more abundant (*p* = 0.018) in AY steers than in AY heifers and was similar (*p* = 0.479) between the two sexes of YL cattle. The relative abundances of *Verrucomicrobia* and *Desulfobacteria* were higher (*p* < 0.05) in YL cattle than in AY cattle, whereas their abundances were unaffected by sex. *Actinobacteria*, *Patescibacteria*, *Fibrobacteres*, *Spirochaetes*, and *Cyanobacteria* presented a similar (*p* > 0.05) relative abundance between both breeds and sexes, although they were considered as dominant phyla.

A total of 18 bacterial genera with an average relative abundance ≥ 0.5% were identified as the dominant genera (Table 6). Significant (*p* < 0.05) interactions of breed and sex were observed for *Succinivibrionaceae UCG-002* and *Prevotellaceae UCG-001*. Multiple comparisons revealed that *Succinivibrionaceae UCG-002* were more relatively abundant (*p* = 0.017) in AY steers than in AY heifers, whereas this genus was no different (*p* = 0.417) between YL steers and YL heifers, while *Prevotellaceae UCG-001* were more relatively abundant (*p* = 0.001) in YL heifers than in YL steers, and the relative abundance of this genus in AY cattle was unaffected (*p* = 0.850) by sex. Regarding the main effects, YL cattle had higher (*p* < 0.05) relative abundances of the *Rikenellaceae RC9 gut group* and *Prevotellaceae UCG-003* and had a lower (*p* = 0.006) relative abundance of *Succinivibrio* compared to AY cattle. In addition, the sex factor only had an impact on the relative abundance of the *Rikenellaceae RC9 gut group*, which was higher (*p* = 0.008) in steers than in heifers.

### 3.5. Correlation Analysis

**Figure 2:**
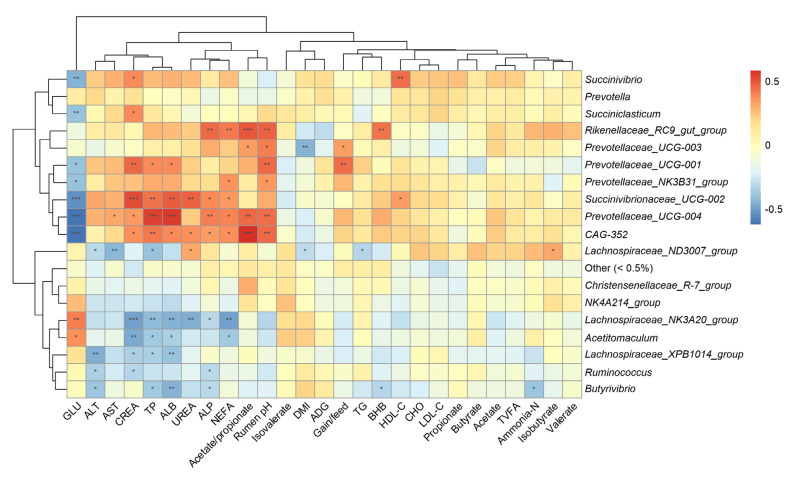
Heatmap of the correlations of genus abundance with fattening performance, serum biochemical parameters, and rumen fermentation. The top and left hierarchical cluster was performed based on the corresponding correlation matrix using the complete linkage method. Lattices are colored based on the corresponding Spearman’s rank correlations. *, **, and *** represent *p* values smaller than 0.05, 0.01, and 0.001, respectively.

A correlation analysis (Figure 2) was conducted to evaluate the genus relationship with fattening performance, serum biochemical parameters, and rumen fermentation. The relative abundance of the *Rikenellaceae RC9 gut group* was positively correlated with NEFA (r = 0.414, *p* = 0.007), ALP (r = 0.456, *p* = 0.003), rumen pH (r = 0.467, *p* = 0.005), and the acetate-to-propionate ratio (r = 0.496, *p* = 0.001). The abundances of *Succinivibrionaceae UCG-002*, *Prevotellaceae UCG-004,* and *CAG-352* were negatively correlated with GLU and positively correlated with CREA, TP, ALB, ALP, and NEFA. The abundances of *Prevotellaceae UCG-003* and *Prevotellaceae UCG-001* were positively correlated with rumen pH (r = 0.413, *p* = 0.014; r = 0.454, *p* = 0.006, respectively) and gain-to-feed ratio (r = 0.336, *p* = 0.032; r = 0.449, *p* = 0.003, respectively).

## 4. Discussion

Small size and slow growth are typical characteristics of southern Chinese indigenous cattle. The adult weights of YL purebred heifers and bulls are only 320 kg and 379 kg, respectively [2]. In the current study, AY cattle had higher initial BW and DMI than YL cattle regardless of sex, which was expected due to the difference in body size between the two breeds. In fact, the improvement in growth performance in AY cattle was mainly inherited from the larger body size of Angus. Interestingly, the breed effects on final BW and ADG differ depending on sex. The growth patterns of bovine tissues, including muscle, fat, and bone, are affected by sired breed and sex [19]. Thus, this interaction effect may be caused by their different tissue growth patterns at the current physiological stage. Besides, YL purebred cattle did not present an inefficiency in feed utilization when compared to crossbred cattle, which may change our view on the fattening benefits of YL purebred cattle. Walker et al. [20] reported that the DMI, ADG, and feed efficiency of purebred Angus fed a high-grain diet without ractopamine were unaffected by sex (steers vs. heifers) except for BW, which is inconsistent with our results. Regardless of these results, we still do not encourage the fattening of heifers, because the population of breeding cows is still one of the important restricting factors for the beef industry development of China.

Measuring the systemic concentrations of serum metabolites could be useful to the overall physiological characterization of animals divergent in breed and sex. Serum metabolites are typically affected by several factors such as diet type, physiological status, and perhaps even animal genetics. In this study, steers had higher concentrations of GLU and BHB and tended to have higher NEFA concentration than heifers regardless of breed. These results are contrary to some previous findings. For instance, Walker et al. [20] found that steers tended to have greater plasma glucose concentration compared to heifers when fed a high-grain diet; Clare et al. [21] found that heifers had higher concentrations of NEFA and BHB and tended to have lower glucose concentration than bulls. Concentrations of NEFA and BHBA are usually used to assess physiological status under a negative energy balance since both rise with fat mobilization and subsequent ketogenesis. Furthermore, those three indicators were correlated with age [22] and even DMI [23]. Thus, the effect of sex on serum biochemical parameters related to energy metabolism could be a combined outcome of different dietary energy intake and physiology statuses. ALP is a biochemical index for diagnosing some diseases, such as liver dysfunction, bone neoplasm, and even cancer [24]. Bone-specific ALP is indicative of bone formation and mobilization [25] and it also reflects phosphorus status in beef cattle [26]. In this study, YL purebred cattle had greater serum ALP concentration than AY cattle. Besides, steers have higher ALP concentration than heifers regardless of breed. Kunkel et al. [27] and Cole et al. [28] have demonstrated that ALP concentration in the blood depends on breed and sex. Inconsistently, their studies revealed that female cattle had higher ALP concentration than male cattle.

Most Chinese indigenous breeds have not undergone long-term commercial breeding and still have relatively poor tolerance to lower rumen pH values caused by a high-grain diet. Decreasing DMI helps to meet the challenge of high-grain diets [3]. When providing a relatively lower energy diet with 64.19% concentration proportions, the DMI, ADG, and rumen pH values of YL steers (*n* = 11) were 6.21 ± 1.19 kg, 516 ± 177 g/d, and 6.64 ± 0.154, respectively (data not shown). This self-protective mechanism of the negative feedback of DMI may be a reason that YL cattle had higher rumen pH values. The interaction of breed and sex was significant for rumen ammonia-N concentration, but no difference was observed for serum UREA concentration. The acetate-to-propionate ratio was different between breeds, which indicates the different rumen fermentation patterns in these two breeds. In addition, we observed that steers had significantly higher isobutyrate and valerate concentrations than heifers, but these findings are inconsistent with a previous study [29]. Although all animals were fed the same diet, their feeding behaviors (e.g., sorting and chewing) may have differed between groups, especially under a high-grain diet, and consequently had a potential impact on rumen fermentation.

Host genetics have a key role in shaping the rumen microbiota. Heterosis comes from parental allelic interactions and different gene expression programs [30] that may alter rumen microbiota. However, there were no differences in rumen bacterial richness and evenness between both breeds and sexes. Furthermore, PCoA and PERMANOVA analysis revealed that the rumen bacterial structure was unaffected by these two factors. Similarly, a recent study reported that bacterial richness and evenness and structure were unaffected by breed when they compared the Chinese Xuanhan indigenous breed and Simmental crossbred cattle fed a diet with a portion of a high-grain content [31], although these two breeds had distinct phenotypic characteristics in terms of growth performance, meat quality, and meat fatty acids [32]. The results are also confirmed by a study [15] comparing Holstein, Jersey, and their hybrid. However, the results of these studies are inconsistent with a study [14] in the *Cervidae* family that revealed the significant differences in rumen bacterial diversity among sika deer (*Cervus Nippon*), elk (*Cervus Elaphus*), and their different hybrid crosses. We suspected the breed effect on rumen bacterial diversity could be explained by differences in genetic distances between species.

Regarding the sex effect, the differences in rumen bacterial diversity (Shannon index) and structure between bulls and heifers were observed in our previous study [29]. A more comprehensive study [10] reported the diversity and abundance of both bacterial communities and archaeal communities were significantly different among bulls, steers, and heifers. Research on the mechanism of sexual differences in rumen microbiota is limited. Some evidence in mice models indicates the differences in gut microbiota between males and females could be partly driven by sexual hormones. For instance, postpubescent mice had more sexual differences in gut microbiota than prepubescent mice, and gonadectomized male mice eliminated the sexual differences [33,34]; testosterone treatment prevented the trends of male mice after gonadectomy [34]. Furthermore, Li et al. [10] suspected that bile acids may mediate the hormone effects on gut microbiota. Meanwhile, animal behavior may be another mediating factor for sexual differences, because males and females may be exposed to different environmental microbes due to different behavior and activities [35]. In this study, steers and heifers were under the same diet and environmental conditions. Thus, gonadectomy may also partly eliminate the sexual differences in rumen bacterial diversity and structure.

Bacteroidetes, Firmicutes, and Proteobacteria represent the majority of rumen bacteria and even intestinal tract of bovine regardless of their different genetic backgrounds [10,31]. We observed the breed interaction with sex for the Proteobacteria phylum abundance, which can be explained by the *Succinivibrionaceae UCG-002* genus, which was more abundant in AY steers than in AY heifers and was unaffected by the sex of YL cattle. *Succinivibrionaceae UCG-002* and *Succinivibrio* were two crucial members of the *Succinivibrionaceae* family in terms of relative abundance. Members of the *Succinivibrionaceae* family were negatively associated with methane emission because its members mainly produce succinate, thereby trapping metabolic hydrogen rather than releasing hydrogen [36,37,38]. In addition, recent studies found the presence of *Succinivibrionaceae UCG-002* and *Succinivibrio* was positively correlated with feed efficiency [39,40]. However, these two genera were unrelated to fattening performance in this study.

The *Rikenellaceae RC9 gut group* belonging to the *Rikenellaceae* family was positively correlated with rumen pH, the acetate-to-propionate ratio, NEFA, BHB, and ALP in this study. The positive relationship between its abundance and dietary fiber has been confirmed by several studies [7,41]. In addition, our previous study [7] found its abundance was positively correlated with rumen pH and acetate and negatively correlated with propionate. A recent study [42] found its abundance was also associated with rumen epithelial morphology. Thus, these findings suggest the presence of the *Rikenellaceae RC9 gut group* has a key role in fiber digestion, rumen fermentation pattern, and rumen epithelial development. However, the relationship between its abundance and serum biochemical parameters related to beta-oxidation of fatty acids (e.g., NEFA and BHB) needs further verification and elaboration.

*Prevotella* as a metabolically and genetically diverse bacterial population is involved in plant cell wall polysaccharides degradation [43], protein catabolism [44,45], etc. Furthermore, its presence impacts amino acid metabolism in host serum [45]. Most of the other members of the *Prevotellaceae* family remain uncultivated. Nevertheless, the metabolic characteristics of the members of the *Prevotellaceae* family should be an important reason for its correlations with rumen pH, fermentation patterns, and serum metabolites. In this study, only two genera, *Prevotellaceae UCG-003* and *Prevotellaceae UCG-001*, were positively correlated with feed efficiency. Inconsistently, recent studies [40,46] suggested the negative correlation of the abundance of *Prevotellaceae* family with feed efficiency. The reason for the inconsistency among these studies may be due to the different animal populations and diets.

The interactions of rumen microbiota with the host have been reviewed in several papers [47,48]. Recent multi-omics studies [12,45] showed that the correlation of rumen microbes with the host’s blood and rumen metabolites is well established. A machine learning approach also suggested that the rumen metabolites of dairy cattle had higher explainability by the core microbiome composition when compared to serum metabolites and milk composition and productions [12]. The differences in explainability by core microbiome composition are well understood. Rumen metabolites are directly produced and utilized by microbes, and only after rumen metabolites interact with gastrointestinal mucosa or are absorbed by the host can they potentially affect serum metabolites and production performance to a certain extent. Future research should use a metabolome approach to extensively determine the complex metabolisms in the rumen, serum, liver, and even beef, and more systematically evaluate their relationship with microbes.

## 5. Conclusions

This is the first study evaluating the interactions of sired breed and progeny sex on rumen bacterial communities and the host’s phenotypic characteristics in beef cattle fed a high-grain diet. The results show that both breed and sex had an impact on fattening performance, serum biochemical parameters, and rumen fermentation. The diversity and structure of rumen bacterial communities were relatively stable under these feeding conditions. Correlation analysis revealed the significant relationships of rumen bacteria with serum biochemical parameters, rumen pH, and rumen fermentation patterns.

## Figures and Tables

**Table 1:** The effects of breed and sex on growth performance of cattle.

Item ^3^	AY ^1^		YL ^2^		Pooled SEM	*p* Value		
	Heifers	Steers	Heifers	Steers		Breed	Sex	B × S
Initial BW (kg)	445	489	274	327	10.2	<0.001	<0.001	0.665
Final BW (kg)	564a	569a	331c	393b	13.8	<0.001	0.020	0.044
DMI (kg/d)	7.99	8.07	4.04	5.51	0.42	<0.001	0.073	0.109
ADG (g/d)	989a	663b	469c	546bc	53.1	<0.001	0.025	0.001
G/F (g/kg)	127	83	122	112	13.6	0.384	0.053	0.232

^1^ AY, Angus × Yiling cattle; ^2^ YL, Yiling cattle; ^3^ BW, body weight; DMI, dry matter intake; ADG, average daily gain; G/F, the ratio of gain and feed. Means followed by different letters in the same row are significant at the *p* < 0.05.

**Table 2:** The effects of breed and sex on serum biochemical parameters of cattle.

Item ^3^	AY ^1^		YL ^2^		Pooled SEM	*p* Value		
	Heifers	Steers	Heifers	Steers		Breed	Sex	B × S
GLU (mmol/L)	2.55	3.05	2.70	3.33	0.284	0.435	0.047	0.857
TG (mmol/L)	0.29	0.28	0.31	0.27	0.023	0.810	0.256	0.448
CHO (mmol/L)	4.08	4.02	4.09	3.93	0.266	0.882	0.685	0.837
NEFA (mmol/L)	0.13	0.19	0.16	0.20	0.025	0.401	0.073	0.711
BHB (mmol/L)	0.13	0.18	0.14	0.20	0.019	0.422	0.007	0.538
HDL-C (mmol/L)	0.29	0.28	0.31	0.27	0.089	0.105	0.596	0.289
LDL-C (mmol/L)	0.93	0.96	1.00	0.92	0.069	0.880	0.745	0.423
CREA (mmol/L)	0.10	0.11	0.10	0.11	0.007	0.777	0.329	0.742
UREA (mmol/L)	4.46	4.62	4.87	4.86	0.234	0.169	0.756	0.727
AST (U/L)	74.8	70.4	56.9	65.3	4.90	0.025	0.685	0.198
ALT (U/L)	23.6	24.6	21.3	24.6	1.57	0.455	0.174	0.479
ALP (U/L)	76.6	102.2	122.0	156.7	12.6	<0.001	0.023	0.722
TP (U/L)	56.0	59.9	59.1	60.2	2.86	0.559	0.381	0.621
ALB (U/L)	32.4	33.4	33.2	33.6	1.20	0.645	0.554	0.793

^1^ AY, Angus × Yiling cattle; ^2^ YL, Yiling cattle; ^3^ GLU, glucose; TG, triglyceride; CHO, cholesterol; NEFA, non-esterified fatty acid; BHB, beta-hydroxybutyrate; HDL-C, high-density lipoprotein cholesterol; LDL-C, low-density lipoprotein cholesterol; CREA, creatinine; UREA, urea; AST, aspartate aminotransferase; ALT, alanine aminotransferase; ALP, alkaline phosphatase; TP, total protein; ALB, albumin.

**Table 3:** The effects of breed and sex on serum rumen fermentation of cattle.

Item ^3^	AY ^1^		YL ^2^		Pooled SEM	*p* Value		
	Heifers	Steers	Heifers	Steers		Breed	Sex	B × S
pH value	6.50	6.65	6.73	6.68	0.062	0.046	0.419	0.128
Ammonia-N (mg/dL)	3.95b	3.72b	3.07b	5.76a	0.554	0.303	0.032	0.012
VFA (mmol/d)								
TVFA	119.8	120.7	105.0	121.0	8.01	0.374	0.302	0.350
Acetate	75.4	75.0	67.5	77.4	5.30	0.606	0.377	0.342
Propionate	25.3	26.4	21.8	23.3	1.82	0.081	0.491	0.908
Isobutyrate	1.05	1.23	0.94	1.70	0.203	0.385	0.027	0.156
Butyrate	14.4	14.1	11.7	14.7	1.00	0.292	0.188	0.120
Isovalerate	2.39	2.53	1.90	2.52	0.202	0.222	0.070	0.250
Valerate	1.34	1.41	1.17	1.51	0.090	0.680	0.033	0.141
Acetate/propionate	2.96	2.90	3.14	3.37	0.145	0.030	0.555	0.334

^1^ AY, Angus × Yiling cattle; ^2^ YL, Yiling cattle; ^3^ VFA, volatile fatty acids; TVFA, total volatile fatty acids. Means followed by different letters in the same row are significant at the *p* < 0.05.

**Table 4:** Alpha diversity index values of ruminal bacteria in different groups.

Item	AY ^1^		YL ^2^		Pooled SEM	*p* Value		
	Heifers	Steers	Heifers	Steers		Breed	Sex	B × S
Observed OTUs	1727	1719	1943	1753	83.6	0.138	0.240	0.279
Good’s coverage (%)	98.3	98.3	98.2	98.4	0.002	0.991	0.477	0.381
Chao1	2374	2378	2677	2387	121	0.206	0.245	0.232
Shannon	7.80	7.86	8.30	7.92	0.167	0.104	0.333	0.186
Simpson	0.977	0.985	0.987	0.981	0.006	0.527	0.848	0.204
PD whole tree	137	139	149	142	4.53	0.102	0.611	0.358

^1^ AY, Angus × Yiling cattle; ^2^ YL, Yiling cattle.

**Table 5:** Relative abundance of the dominant phylum with an average relative abundance ≥ 0.1%.

Item	AY ^1^		YL ^2^		Pooled SEM	*p* Value		
	Heifers	Steers	Heifers	Steers		Breed	Sex	B × S
*Bacteroidetes*	63.6	60.1	64.6	56.0	3.57	0.677	0.100	0.482
*Firmicutes*	30.5	29.8	29.3	38.6	3.74	0.320	0.260	0.187
*Proteobacteria*	2.91b	6.69a	3.24b	2.09b	1.11	0.063	0.246	0.033
*.Actinobacteria*	0.956	0.888	0.369	0.592	0.345	0.209	0.824	0.676
*Patescibacteria*	0.600	0.637	0.463	0.692	0.129	0.754	0.309	0.463
*Fibrobacteres*	0.287	0.467	0.524	0.302	0.120	0.766	0.864	0.103
*Spirochaetes*	0.339	0.360	0.461	0.321	0.070	0.558	0.396	0.255
*Verrucomicrobia*	0.226	0.300	0.371	0.401	0.0470	0.013	0.278	0.641
*Desulfobacteria*	0.199	0.226	0.268	0.328	0.0273	0.003	0.118	0.553
*Cyanobacteria*	0.173	0.299	0.201	0.255	0.0598	0.890	0.140	0.555
Others	0.203	0.271	0.208	0.389	0.0453	0.178	0.009	0.218

^1^ AY, Angus × Yiling cattle; ^2^ YL, Yiling cattle. Means followed by different letters in the same row are significant at the *p* < 0.05.

**Table 6:** Relative abundance of the dominant genus with an average relative abundance ≥ 0.5%.

Item	AY ^1^		YL ^2^		Pooled SEM	*p* Value		
	Heifers	Steers	Heifers	Steers		Breed	Sex	B × S
*Prevotella*	46.4	43.5	42.4	36.5	4.13	0.189	0.296	0.721
*Christensenellaceae R-7 group*	3.41	3.87	3.72	6.98	1.05	0.112	0.086	0.190
*Ruminococcus*	4.10	3.19	3.48	3.67	0.634	0.910	0.574	0.392
*Succiniclasticum*	3.67	3.39	3.59	2.85	0.569	0.586	0.376	0.688
*NK4A214 group*	2.21	2.58	2.59	4.63	0.671	0.080	0.082	0.222
*Rikenellaceae RC9 gut group*	1.98	2.72	2.75	4.36	0.421	0.007	0.008	0.304
*Succinivibrionaceae UCG-002*	1.46b	4.51a	2.33ab	1.27b	0.885	0.191	0.269	0.026
*Prevotellaceae UCG-003*	1.85	1.93	3.01	2.36	0.219	0.001	0.206	0.105
*Prevotellaceae UCG-001*	1.83b	1.76b	2.59a	1.32b	0.249	0.532	0.011	0.021
*Lachnospiraceae NK3A20 group*	1.04	1.38	0.92	2.38	0.533	0.416	0.100	0.299
*Lachnospiraceae ND3007 group*	0.607	0.773	1.018	0.848	0.289	0.408	0.995	0.566
*Lachnospiraceae XPB1014 group*	0.575	0.722	0.636	1.287	0.246	0.212	0.114	0.313
*Prevotellaceae NK3B31 group*	0.591	0.626	0.947	0.606	0.151	0.272	0.318	0.221
*Prevotellaceae UCG-004*	0.533	0.542	1.020	0.474	0.200	0.304	0.188	0.174
*Acetitomaculum*	0.502	0.530	0.367	1.034	0.211	0.388	0.108	0.139
*Butyrivibrio*	0.639	0.699	0.458	0.544	0.124	0.182	0.560	0.917
*CAG-352*	0.504	0.543	0.840	0.399	0.127	0.453	0.123	0.067
*Succinivibrio*	0.717	0.886	0.210	0.142	0.215	0.006	0.817	0.584
Others (<0.5%)	27.3	25.8	27.2	28.3	1.78	0.517	0.924	0.451

^1^ AY, Angus × Yiling cattle; ^2^ YL, Yiling cattle. Means followed by different letters in the same row are significant at the *p* < 0.05.

## Data Availability

The datasets generated for this study can be found in online repositories. The name of the repository (NCBI) and accession number (PRJNA769506) can be found in the following link: https://www.ncbi.nlm.nih.gov/bioproject/PRJNA769506, accessed on 8 October 2021.
